# Emerging Roles of ADAMTSs in Angiogenesis and Cancer

**DOI:** 10.3390/cancers4041252

**Published:** 2012-11-29

**Authors:** Saran Kumar, Nithya Rao, Ruowen Ge

**Affiliations:** Department of Biological Sciences, National University of Singapore, Singapore 117543, Singapore; E-Mails: saran@nus.edu.sg (S.K.); g0801253@nus.edu.sg (N.R.)

**Keywords:** angiogenesis, cancer, ADAMTS, metalloproteinase, proteoglycanase, tumorigenesis, metastasis

## Abstract

A Disintegrin-like And Metalloproteinase with ThromboSpondin motifs—ADAMTSs—are a multi-domain, secreted, extracellular zinc metalloproteinase family with 19 members in humans. These extracellular metalloproteinases are known to cleave a wide range of substrates in the extracellular matrix. They have been implicated in various physiological processes, such as extracellular matrix turnover, melanoblast development, interdigital web regression, blood coagulation, ovulation, *etc*. ADAMTSs are also critical in pathological processes such as arthritis, atherosclerosis, cancer, angiogenesis, wound healing, *etc*. In the past few years, there has been an explosion of reports concerning the role of ADAMTS family members in angiogenesis and cancer. To date, 10 out of the 19 members have been demonstrated to be involved in regulating angiogenesis and/or cancer. The mechanism involved in their regulation of angiogenesis or cancer differs among different members. Both angiogenesis-dependent and -independent regulation of cancer have been reported. This review summarizes our current understanding on the roles of ADAMTS in angiogenesis and cancer and highlights their implications in cancer therapeutic development.

## 1. Introduction

**Figure 1:**
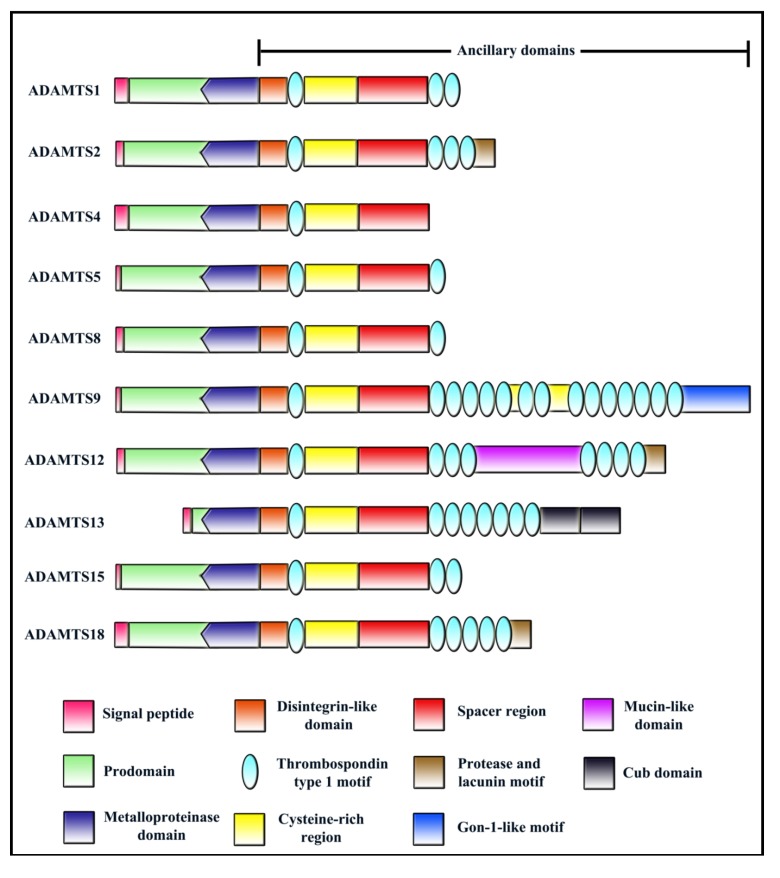
Domain structure of ADAMTS members with reported roles in angiogenesis/cancer.

The ADAMTS family of secreted metalloproteinases is characterized as having one or more Thrombospondin Type 1 repeat (TSR) domains in their ancillary regions. All ADAMTS members have the following domain structure from the *N*-terminus: (i) a signal peptide that helps in directing ADAMTS to the secretory pathway; (ii) a prodomain which play a part in maintaining enzyme latency (with the exception of ADAMTS9 [1] and ADAMTS13 [2]); (iii) a zinc binding metalloproteinase domain; (iv) a disintegrin-like domain; (v) a central TSR; (vi) a cysteine rich domain; (vii) a spacer domain; (viii) variable number of *C*-terminal TSRs (Figure 1). Readers may refer to several excellent reviews on ADAMTS family published previously for more information [3,4,5,6,7,8,9].

ADAMTS members can be classified into subgroups based on its functions such as pro-collagen processing (ADAMTS2, ADAMTS3 and ADAMTS14), proteoglycan degradation (ADAMTS1, ADAMTS4, ADAMTS5, ADAMTS8, ADAMTS9 ADAMTS15 and ADAMTS20), blood coagulation (ADAMTS13), and cartilage oligomeric matrix protein (COMP) degradation (ADAMTS7 and ADAMTS12). Up to now, functions of several orphan ADAMTS members are still unknown including ADAMTS6, ADAMTS16, ADAMTS17, ADAMTS18 and ADAMTS19. Although ADAMTSs have been extensively studied for its catalytic function, more and more reports on their role in angiogenesis and cancer have emerged in recent years, with 10 out of 19 members reported so far. Many ADAMTS members have been shown to negatively regulate angiogenesis in the past decade, thus making it into the list of endogenous angiogenesis inhibitors [6]. Interestingly, ADAMTS13 is the only member in this family to exhibit both pro- and anti-angiogenic activities [10]. It is also interesting to note that even though these 10 ADAMTS—ADAMTS1, ADAMTS2, ADAMTS4, ADAMTS5, ADAMTS8, ADAMTS9, ADAMTS12, ADAMTS13, ADAMTS15, and ADAMTS18 have emerged as important players in angiogenesis and/or cancer, their mechanisms of action are not shared by all family members. Notably, not all ADAMTS members regulate cancer through influencing angiogenesis. Some ADAMTS members have been well established as tumor suppressors without any reported roles in angiogenesis. This review summarizes the large amount of reported studies with regard to roles of ADAMTSs in angiogenesis and cancer and discusses their significance in cancer therapeutic development.

## 2. ADAMTS1

ADAMTS1/METH-1 was first discovered in mouse by differential display from colon 26 adenocarcinoma cachexigenic tumor cell lines [11]. Human *ADAMTS1* was isolated from heart and lung cDNA libraries during a search for novel genes containing the anti-angiogenic TSR domain [12]. The human gene is located on chromosome 21q21.2.

### 2.1. Structure and Processing

*ADAMTS1* is a single copy gene in the human genome translating into a 110 kDa protein. It possesses a multi-domain structure—putative signal peptide, prodomain, zinc metalloproteinase, disintegrin-like region, central TSR, spacer and 2 *C*-terminal TSR domains (Figure 1) [13]. The human ADAMTS1 has 83.4% amino acid identity with the mouse homologue [12]. A potential furin cleavage site (RKKR) lies between prodomain and metalloproteinase domain. The 110 kDa full protein is processed to mature 85 kDa most likely by cleavage at RKKR site and a more abundant 67 kDa form by a second processing event [12]. Post-translationally, ADAMTS1 is modified by glycosylation at four putative *N*-linked glycosylation sites [13].

### 2.2. Regulation and Expression

Embryonic expression pattern of *Adamts1* in developing mouse embryo from day 10 (E10) to E18 revealed detectable levels of *Adamts1* transcripts in yolk sac, placenta, brain, heart, lung, limb bud, liver, spleen, and kidney, while much lower levels were observed in adult. Expression is predominantly restricted to the epithelium. Expression was also detected in tunica media of the aorta, pulmonary, and hepatic vessels [14].

All human tissues showed *ADAMTS1* mRNA expression with high expression in heart, adrenal, placenta, skeletal muscle, thyroid and stomach as well as embryonic kidney. Expression was also detected in dermal fibroblasts and low level expression found in endometrial, stromal, vascular smooth muscle cells (VSMC) and some endothelial cells (ECs) [12]. In addition, strong *ADAMTS1* expression has also been found in luminal epithelial cells in benign prostate glands [15].

*ADAMTS1* expression could be regulated by cytokines and inflammatory stimulators such as interleukin-1 (IL-1), bacterial lipopolysaccharide (LPS) and tumor necrosis factor—α (TNF-α). Induction by IL-1 *in vitro* and enhanced mRNA expression in kidney and heart following LPS administration *in vivo* suggests the involvement of ADAMTS1 in inflammatory processes [13]. In addition, TNF-α was also found to cause an up-regulation of *ADAMTS1* mRNA levels in Cos-7 cells [16]. However in case of IL-1β stimulation, transcriptional down-regulation of *ADAMTS1* was observed in chondrosarcomas [17].

Post-transcriptionally, the 3'UTR of *ADAMTS1* regulates its mRNA stability possibly via multiple AUUUA motifs located in its 3'UTR [16]. A yeast two-hybrid screen exploring for potential interaction partners of ADAMTS1 identified fibulin-1 as a new regulator of ADAMTS1-mediated proteoglycan degradation. Fibulin-1 enhances ADAMTS1’s capacity to cleave aggrecan and may have a relevance in the morphogenesis of kidney epithelium and other functions [18].

### 2.3. Function

ADAMTS1 is necessary for normal growth, structure and function of kidneys, adrenal glands and female reproductive organs as *ADAMTS1* null mice were observed to be smaller than wild-type litter mates, exhibit urological abnormalities, adrenal gland abnormalities and abnormalities in female genital organs and impaired fertility in females (Table 1) [19,20,21]. ADAMTS1 as a metalloproteinase has multiple extracellular matrix proteoglycan substrates such as aggrecan, which it cleaves at multiple sites, versican V1, nidogen-1 and 2 and tissue factor pathway inhibitor-2 (TFPI-2), which may have implications in ECM remodelling in pathological conditions such as atherosclerosis or tumor progression [22,23,24,25]. ADAMTS1 has also been implicated in wound healing [26].

**Table 1:** Alternative names, substrates and knockout phenotypes of ADAMTS members implicated in angiogenesis and cancer.

Protein	Alternative Names	Substrates	Knockout phenotype
**ADAMTS1**	METH-1, aggrecanase-3	Aggrecan [22]	a. Growth retardation, changes in kidney structure and impaired female fertility [19,20].
		Versican V1 [23]	
		Nidogen 1 & 2	
		Tissue Factor Pathway Inhibitor-2 [25]	b. No effect on aggrecan turnover [27].
**ADAMTS2**	PCINP	Procollagens type I, II, III and V [28,29,30]	a. Fragile skin and male sterility [31].
**ADAMTS4**	aggrecanase-1, KIAA0688	Aggrecan [32,33]	a. Phenotypically normal, no protection against aggrecan degradation [38].
		Versican [23]	
		Brevican [34]	
		Matrilin [35] Hevin [36] Reelin [37]	
**ADAMTS5**	aggrecanase-2, ADAMTS11, *Implantin*	Aggrecan [39], Versican [40], Brevican [41], Neurocan [42].	a. Normal lifespan, healthy and fertile [43,44]. Syndactyly with a penetrance of 44% [45].
			b. Reduced cartilage degradation in a mouse model of osteoarthritis [43].
			c. Delayed wound healing due to aggrecan deposition [46].
			d. Double knockout (*Adamts4^−^^/^^−^*, *Adamts5^−^^/^^−^*)—Normal, healthy and fertile [47,48]. Reduced body weight in females compared to wild type. Protected against arthritis [47].
			e. Double knockout (*Adamts5^−^^/^^−^*, Adamts20^−/−^)—No gross abnormalities, soft tissue Syndactyly [45].
**ADAMTS8**	METH-2	Aggrecan [49].	-
**ADAMTS9**	KIAA1312	Aggrecan [50],	a. Embryonically lethal [51].
		Versican [50].	b. Haploinsufficiency causes increased angiogenesis [52], cardiac and aortic anomalies [53].
			c. *Adamts5*^−/−^, *Adamts9*^+/−^: soft tissue Syndactyly [45].
**ADAMTS12**	-	COMP [54].	a. Phenotypically normal and fertile [56].
		Aggrecan [55]	b. Elevated angiogenesis [56].
			c. Severe inflammation [57]
**ADAMTS13**	vWFCP	vWF [58].	a. Viable and fertile [59,60].
**ADAMTS15**	-	Aggrecan [61],	Not known
		Versican [62].	
**ADAMTS18**	-	Not known	Not known

### 2.4. Angiogenesis and Cancer

Vazquez *et al.* first identified ADAMTS1/METH1 as a novel anti-angiogenic protein (Table 2) [12]. ADAMTS1 suppresses EC proliferation in a cell-specific, dose-dependent manner via interruption of VEGFR2 signalling [12,63]. It also disrupted growth-factor induced angiogenesis *in vivo* both in CAM (chorioallantoic membrane) assay and cornea pocket assay, more potently than TSP-1 or endostatin [12].

**Table 2:** ADAMTS role in angiogenesis.

Protein	Dependence on catalytic activity	Involvement of TSRs	Role in Angiogenesis
**ADAMTS1**	Yes (Pro-angiogenic) [64]	Yes (Anti-angiogenic) [66]	**Anti-angiogenic **
	Yes (Anti-angiogenic) [63]		- Suppresses EC proliferation in a cell-specific, dose dependent manner [12,63]
	No (Anti-angiogenic) [65]		- Disrupts growth factor induced angiogenesis *in vivo* in a CAM model and matriplug assay [12].
			- Suppresses tumor angiogenesis in T47D human breast carcinoma [63]
			- Regulates angiogenesis in ischemic myocardium [67]
			- Suppresses tumor angiogenesis in HT-1080, DU145 and CHO-K1 tumors [65].
			- Alters blood vessel morphology in prostate tumors [68]
			- Induced by VEGF in ECs and ischemia induced retinal neovascularization [69]
			**Pro-angiogenic**
			- Induction of ADAMTS1 to degrade basement membrane versican in VEGF-induced pathological angiogenesis [64].
			- Promotes tumor angiogenesis in TA3 mammary carcinoma and Lewis lung carcinoma [66]
			- Induces endothelial-like phenotype in plastic tumor cells [70]
**ADAMTS2**	No (Anti-angiogenic) [71]	Yes (Anti-angiogenic) [71]	**Anti-angiogenic**
			- Suppressed VEGF-stimulated EC proliferation in a cell-specific manner, induces apoptosis and inhibits capillary network formation of HUVEC [71].
			- Increased blood vessels *in vivo* in a CAM model in *ADAMTS2* knockout mice [71].
			- Suppressed tumor angiogenesis in ADAMTS2 overexpressing tumors [71].
**ADAMTS4**	Yes (Pro-angiogenic) [72]	Yes (Anti-angiogenic) [72]	**Anti-angiogenic**
			- Anti-angiogenic peptide from ADAMTS4 TSR suppresses EC proliferation and VEGF-induced HUVEC migration [73].
			- Truncated ADAMTS4 fragment inhibits HuDMEC differentiation and migration in a scratch wound healing assay [74].
			- ADAMTS4 *C*-terminal ancillary regions inhibit tumor angiogenesis [72].
			**Pro-angiogenic**
			- Full-length ADAMTS4 promotes tumor angiogenesis [72].
**ADAMTS5**	No (Anti-angiogenic) [75]	Yes (Anti-angiogenic) [75,76]	**Anti-angiogenic**
			- ADAMTS5 is anti-angiogenic *in vitro* and *in vivo* [75,76].
**ADAMTS8**	Not known	Yes (Anti-angiogenic) [12]	**Anti-angiogenic**
			- Inhibits EC proliferation in a cell-specific reversible manner* in vitro* [12].
			- Disrupts growth factor induced angiogenesis *in vivo* in a CAM model and matriplug assay [12].
**ADAMTS9**	Yes (Anti-angiogenic) [52]	No (Anti-angiogenic) [52]	**Anti-angiogenic**
			- Knockdown of ADAMTS9 in cultured ECs suppresses *in vitro* capillary network formation and migration [52].
			- Increased corneal neovascularization and tumor vascularization in *in vivo*^+/−^ mice compared to the wild type mice [52].
			- Suppresses oesophageal and nasopharyngeal carcinoma angiogenesis [77]
**ADAMTS12**	No (Anti-angiogenic) [56]	Yes (Anti-angiogenic) [55]	**Anti-angiogenic**
			- Inhibits capillary network formation by BAE-1 cells in 3D collagen gels [55].
			- *Adamts12*^−/−^ mice showed increased sprout density in both *ex vivo* and *in vivo* models of angiogenesis [56].
**ADAMTS13**	Not known	Yes (Anti-angiogenic) [10]	**Anti-angiogenic**
			- ADAMTS13 inhibits VEGF-mediated angiogenesis-mediated HUVEC proliferation, migration and capillary network formation [10].
			**Pro-angiogenic**
			- Full-length ADAMTS13 promoted HUVEC tube formation, induces EC proliferation and migration *in vitro* [10].
**ADAMTS15**	Not known	Not known	Not known
**ADAMTS18**	Not known	Not known	Not known

Two possible mechanisms were revealed for its anti-angiogenic function. First is via the release of anti-angiogenic peptides from TSP-1 and TSP-2, suggesting that the catalytic activity of protein is important [78]. The second possible mechanism is via sequestration of vascular endothelial growth factor (VEGF) by means of an interaction between the heparin binding domain in VEGF and the *C*-terminal spacer domain plus TSRs. VEGF sequestration results in the loss of signalling via VEGFR2 receptor and consequent suppression of EC proliferation [79]. However, in a mouse model of ischemia-induced retinal neovascularization, along with an increase in VEGF levels, *ADAMTS1* mRNA and protein levels also increased. VEGF has also been shown to increase ADAMTS1 levels in EC in a protein kinase C (PKC) dependent fashion, suggesting a feedback inhibition loop [69]. In another study by Fu *et al.* VEGF overexpression-induced pathological angiogenesis may involve the induction of ADAMTS1 by VEGF which subsequently causes degradation of basement membrane versican [64]. Thus, it seems that in conditions of VEGF-induced pathological angiogenesis, ADAMTS1 may be co-induced, wherein it might be performing contrasting functions. In some cases, acting via metalloproteinase domain, ADAMTS1 might aid angiogenesis, while in other cases, acting via TSR domains, it inhibits angiogenesis.

ADAMTS1 has also been linked to ischemic disease, where the process of collateral vessel development (angiogenesis) after myocardial infarction is crucial. *ADAMTS1* mRNA levels were found to be increased in ischemic myocardium, along with VEGF, where it could have a potential role as a regulator of angiogenesis via binding to VEGF [67]. The same group also showed that *ADAMTS1* was a hypoxia inducible gene with transient up-regulation of *ADAMTS1* transcript in ECs by HIF-1 [80]. This transient up-regulation of *ADAMTS1* was indicative of an induction only under acute hypoxic conditions. Taking their cue from this observation, Cilek *et al*. have developed an *ADAMTS1* promoter driven gene expression system, that could be induced by acute hypoxia both *in vitro* and *in vivo* [81]. This system could prove as promising strategy for gene therapy in future.

Reports of differential expression of *ADAMTS1* in several cancer types strongly suggest a role for ADAMTS1 in cancer. High level *ADAMTS1* expression was noted in HeLa (cervical carcinoma) and G631 (melanoma) [12]. Four out of six pancreatic cancer cell lines also showed ADAMTS1 expression, in addition to both noncancerous and pancreatic cancer tissues [82]. However, 30% of non-small cell lung carcinoma (NSCLC) cell lines showed a down-regulation of *ADAMTS1* [83]. *ADAMTS1* expression was also markedly low in human prostate cancer cells and in patients with metastases from hormone refractory prostate tumors [15].

In line with the differential expression of *ADAMTS1* in several cancer types, ADAMTS1 seems to promote tumor growth and metastasis in some cancers while in others its expression is down-regulated implicating a tumor suppressor role (Table 3). Several reports suggest a role for ADAMTS1 in promoting tumor cell invasion. In endometrial cancers, elevated ADAMTS1 level via prostaglandin F-prostanoid (PGF2α-FP) signalling through a calmodulin-NFAT (nuclear factor of activated T-cells)-dependent pathway promotes epithelial cell invasion through ECM and inhibits EC proliferation [84]. Recently, Ricciardelli *et al*. have shown that ADAMTS1 may promote mammary tumor growth and progression to metastasis using a PyMT model. *Adamts1*^−/^^−^/PyMT mice displayed significantly reduced mammary tumor and lung metastatic tumor burden and increased survival, compared with their wild-type and heterozygous littermates [85]. Interestingly the anti-angiogenic action ADAMTS1 was not a major contributing factor to total tumor angiogenesis as there was no significant increase in blood vessel density in *ADAMTS1* null tumors. In bone metastasis, acting as a sheddase, ADAMTS1 promotes the release of membrane bound epidermal growth factor (EGF)-like growth factors, including amphiregulin (AREG), heparin-binding EGF (HB-EGF), and transforming growth factor α (TGFα) from tumor cells [86]. Another interesting example of ADAMTS1 sheddase activity is the differential control of metastatic disease via regulation of angiogenesis. ADAMTS1 cleaves matrix-bound TSP-1, releasing the anti-angiogenic 3TSR region. Recently Lee *et al*. have shown that TSP-1 is processed more efficiently in liver compared to lung, thus leading to a marked reduction in liver metastases but not lung [87]. In NSCLC however, *ADAMTS1* is down-regulated via promoter methylation and thus could be acting as a tumor suppressor in this cancer [83]. Surprisingly, in pancreatic cancer, although *ADAMTS1* levels were much lower in comparison to noncancerous pancreatic tissue, patients with lymph node metastasis or severe retroperitoneal invasion exhibited higher levels of *ADAMTS1* expression and poorer recovery rates [82]. In yet another scenario, ADAMTS1 increased fibrosarcoma tumor growth rate in an angiogenesis-independent manner, with tumor cells acquiring a endothelial-cell like phenotype possibly through a vascular mimicry mechanism [70].

In prostate cancer, ADAMTS1 plays intricate and complex roles linked to the ability of tumors to respond to the hormone androgen. On one hand, ADAMTS1 is lowered in prostate cancer and may be involved in early stages of prostate cancer development, with low levels of ADAMTS1 associated with high microvessel density and metastasis [15]. On the other hand, ADAMTS1 acts as a tumor promoting factor in androgen-dependent LnCAP tumors, as down-regulation of ADAMTS1 results in reduced tumor growth in these tumors. However, in androgen-independent LnCAP-19 tumors, expression of ADAMTS1 delays tumor establishment [68]. Interestingly, ADAMTS1 expression levels correlate with altered blood vessel morphology with low level ADAMTS1 linked with small diameter vessels while high level ADAMTS1 associated with larger diameter blood vessels [68].

It is well established that tumor cells and stromal cells signal each other in the tumor microenvironment. ADAMTS1 is also involved in such a tumor-stromal interaction. Utilizing a bronchial epithelial tumor cell line (BZR), Rocks *et al.* showed that expression of ADAMTS1 in tumor cells promotes tumor growth through induction of a stromal reaction characterized by myofibroblast infiltration and excessive matrix deposition [88]. These stromal reaction features are not observed in tumors derived from cells overexpressing a catalytically inactive mutant of ADAMTS1. On the other hand, breast cancer cells induce ADAMTS1 secretion from cancer associated fibroblasts when co-cultured together, resulting in cancer cell invasion [89]. Thus it could be seen that ADAMTS1 could both induce as well as be induced by stromal components, exhibiting an excellent example of tumor-stroma crosstalk.

In other experimental tumor models, ADAMTS1 also presented a complex and often contradicting role in different studies. For example, expression of human ADAMTS1 was shown to exert an anti-tumor effect on T47D breast cancer cells. Both the catalytic site mutant as well as the *C*-terminal regions of the protein lost its anti-tumor activity in xenograft tumor assays, indicating the importance of catalytic activity for maintaining the anti-tumor activity of this protein [63]. However Kuno *et al.* using mouse Adamts1 and Chinese hamster ovary (CHO) cells demonstrated that the *C*-terminal ancillary region alone inhibited tumor growth and both the TSR domains as well as the spacer domain are important for mediating this function [90]. In addition, a recent study by Obika *et al.* showed that ADAMTS1 suppressed subcutaneous tumor growth and inhibited tumor angiogenesis in three different tumor cell lines HT-1080, DU-145 and CHO-K1 and this anti-tumorigenic/anti-angiogenic effect is independent of its catalytic activity [65].

**Table 3:** Expression and regulation of ADAMTSs in various cancers.

Protein	Cancer type	Regulation
**ADAMTS1**	Lung cancer	Down-regulation of *ADAMTS1* mRNA in NSCLC cell lines and epigenetic regulation via hypermethylation of its promoter [83]
	Pancreatic cancer	*ADAMTS1* mRNA expression significantly lower in pancreatic cancer compared to noncancerous pancreas [82].
		Enhanced expression of *ADAMTS1* mRNA in lymph node metastasis or severe retroperitoneal invasion [82].
	Prostate cancer	Markedly low protein levels in prostate cancer cells [15].
	Chondrosarcoma	Transcriptional up-regulation in response to TNF-α [16].
**ADAMTS2**	Osteosarcoma	8-fold increase in *ADAMTS2* mRNA levels [28].
**ADAMTS4**	Breast cancer	Enhanced mRNA expression in breast cancer compared to normal breast tissue [91].
	Head and neck squamous cell carcinoma	Enhanced expression of *ADAMTS4* mRNA [92].
	Glioblastoma	Increased expression of *ADAMTS4* mRNA [93].
	Ewings sarcoma	Enhanced protein levels serving as a tumor-specific marker [94].
**ADAMTS5**	Breast carcinoma	Down-regulation of *ADAMTS5* transcript [91].
	Colorectal cancer	Epigenetically silenced by promoter methylation [95].
	Prostate cancer	Down-regulation of *ADAMTS5* mRNA in prostate cancer cell lines [42].
	Glioblastoma	Overexpression of *ADAMTS5* mRNA and protein [41,93].
**ADAMTS8**	Lung cancer	Down-regulation of *ADAMTS8* at the mRNA level in NSCLC [96].
		Down-regulation due to epigenetic silencing [97].
	Brain	Down-regulation due to promoter hypermethylation [98].
**ADAMTS9**	Breast carcinoma	Down-regulation of *ADAMTS9* transcript [91].
	Esophageal squamous cell carcinoma	Hypermethylation of *ADAMTS9* in esophageal tumors [99].
	Nasopharyngeal carcinoma	Promoter hypermethylation and association of lower levels of ADAMTS9 protein with lymph node metastasis in NPC [100].
	Gastric cancer	Epigenetic silencing by promoter hypermethylation [101].Inhibition through Akt/mTOR pathway [102].
	Pancreatic and colorectal cancer	Epigenetic silencing by promoter hypermethylation [101].
**ADAMTS12**	Colorectal cancer	Epigenetic silencing by promoter hypermethylation [103].
**ADAMTS13**	Prostate, renal, testicular, head and neck squamous, colorectal, rectal, NSCLC, gastric, melanoma, adenocarcinoma and breast carcinoma.	Correlation of presence or absence of tumor metastasis with lower or higher vWF cleaving respectively [104].
	Colon cancer, leukemia, multiple myeloma, breast, stomach cancer, non-Hodgkin’s lymphoma	Decreased activity of ADAMTS13 in plasma of malignant patients [105].
	Brain and prostate cancers	Mild reduction in ADAMTS13 activity but no correlation with malignancy and metastasis [106].
**ADAMTS15**	Colorectal and pancreatic cancer	Inactivation and loss of normal function of the protein due to somatic mutations [107,108].
	Colorectal cancer	Loss of heterozygosity in *ADAMTS15* locus [109].
	Breast cancer	Grade-specific down-regulation of *ADAMTS15* transcript in breast cancer [91].
	Prostate cancer	Down-regulation of *ADAMTS15* mRNA linked to poor prognosis in prostate cancer [42].
**ADAMTS18**	Breast cancer	Down-regulation of *ADAMTS18* transcript [91].
	Pancreatic, gastric and colorectal cancers	Hypermethylation of *ADAMTS18* promoter [110]
	Kidney and colorectal cancers	Inactivation of *ADAMTS18* via somatic mutations [111].
	Melanoma	Somatic mutations in *ADAMTS18* linked to higher transformation ability and increased metastases *in vivo* [112].

Kuno *et al.* also showed that both the full-length protein as well as the *C*-terminal regions exhibited an anti-metastatic activity [90]. In contrast, Liu *et al.* reported that full-length ADAMTS1 increased the number of lung metastases while *C*-terminal regions of the protein exhibited anti-metastatic activity using two tumor models—TA3 mammary carcinoma and Lewis lung carcinoma [66]. Notably, Liu *et al.* showed that a catalytic active site mutant of ADAMTS1 inhibited metastasis instead, indicating that catalytic activity is important for the pro-metastatic activity of ADAMTS1. The primary mechanism for eliciting this function being the shedding of trans-membrane precursors heparin-binding epidermal growth factor (HB-EGF) and amphiregulin that activate the EGFR receptor, ultimately promoting invasion [66]. Most importantly, this work convincingly demonstrated that while the full-length ADAMTS1 is pro-metastatic, auto-catalytic fragments of ADAMTS1 composed of *N*-terminal and *C*-terminal cleavage fragments containing TSR domains act as anti-metastatic molecules in the same tumors. Thus, they proposed that the proteolytic status of ADAMTS1 determines its net effect on tumor metastasis.

It is likely that the different proteolytic status of ADAMTS1 protein may not be the only reason for the conflicting roles reported on ADAMTS1 in primary tumor growth and metastasis. Conceivably, the local microenvironment of the particular tumor, the level of *ADAMTS1* expression, the type of substrates present in the local ECM, and the tissue-specific characteristics of tumor cell types all contribute to the net influence of ADAMTS1 in particular types of tumor. Moreover, ADAMTS1 has demonstrated angiogenesis dependent and angiogenesis-independent effects on tumor growth and metastasis from different studies. Thus, further work is required to clarify this discrepancy; whether the cleavage of ADAMTS1 is regulated and if yes, how it is regulated; what are the catalytic-dependent and catalytic-independent functions of ADAMTS1 in cancer; what are the interplay between ADAMTS1 and other ADAMTS members in cancer.

## 3. ADAMTS2

ADAMTS2 was identified as a pro-collagen I *N*-proteinase, a neutral Ca^2+^ dependent proteinase that specifically cleaves type I and type II collagens. The enzyme is located at the long arm of chromosome 5 at its terminus. The enzymatic activity was first detected in extracts of normal calf tissues as early as 1971 [113]. Isolated from bovine skin with a molecular mass of around 110 kDa, the protein is often found to occur in multimeric complexes [114]. It has chiefly been studied for its involvement in dermatosparaxis in cows and sheep and type VIIC Ehlers Danlos syndrome in humans, both connective tissue skin disorders [115,116,117].

### 3.1. Structure and Processing

Full-length human pro-ADAMTS2 has a molecular size of 150 kDa. It shares the typical domain structure with other family members comprising of a 36 amino acid signal peptide, a prodomain, a metalloproteinase domain with a Zn^2+^ binding site followed by a Met-turn, a characteristic of clan MB of metallopeptidases, a disintegrin-like domain containing a RGD sequence, a potential integrin binding site (Figure 1) [118]. The protein also has four TSR repeats, one centrally located and three in *C*-terminus of the protein. Between the two groups of TSR repeats lay a cysteine-rich region and a spacer domain. A protease and lacunin motif is located beyond the *C*-terminal TSR repeats, a domain reported to be associated with epithelial remodelling [119].

ADAMTS2 is released from enzyme latency by cleavage at potentially two consensus sequence sites RTRR and RRRMRR, known to be specific to mammalian subtilisins such as furins. The mature processed form thus corresponds to a size of 132 kDa. Another 141 kDa form resulting from cleavage at the more *N*-terminal furin cleavage consensus site appears to be a solely intracellular form [28]. Post translationally, ADAMTS2 could possibly be glycosylated as seen from the presence of 9 potential glycosylation sites (NXT/S), mostly in the *C*-terminus of the protein [28,118].

### 3.2. Expression and Regulation

*ADAMTS2* mRNA was expressed at high levels in type-I collagen rich tissues such as skin, bones, tendons and aorta and trace amounts in brain and thymus. In line with this observation enzyme activity also paralleled the mRNA expression of *ADAMTS2* [118].

ADAMTS2 has been shown to exist in two forms: a long form that closely resembles the bovine enzyme and a short form that lacks the entire *C*-terminus region of the protein and thus most of the TSR repeats and glycosylation sites, a consequence of using a polyadenylation signal within an intronic sequence of the long form pre-mRNA. A physiological role for this shorter form is suggested by the abundance of this form in skin and cultured fibroblasts, although there is no detailed study [29]. 

Besides this, *ADAMTS2* is also regulated by TGF-β1 at transcriptional level, which induces an 8-fold increase in *ADAMTS2* mRNA levels in MG-63 human osteosarcoma cells in a dose- and time-dependent manner, without affecting RNA stability [28]. *ADAMTS2* is subjected to multiple mutations causing type VIIC Ehlers Danlos syndrome or dermatosparaxis in cows and sheep [29,120,121,122]. Furthermore, Li *et al*. have also shown that transgenic mice with inactivated alleles of *Adamts2* developed fragile skin and male sterility [31].

### 3.3. Function

ADAMTS2’s major function is cleavage of amino propeptides of type I, II, III and V pro-collagens, which has been extensively characterized (Table 1) [28,29,30]. It has been suggested that ADAMTS2 may have other functions besides processing of pro-collagen such as proteolytic processing of other substrates, maturation of spermatagonia or even involvement in angiogenesis [28,31,118].

### 3.4. Angiogenesis and Cancer

ADAMTS2 shows the presence of CSVTCG motif within its TSR repeats. The second TSR domain of TSP-1 has been previously shown to inhibit angiogenesis via this motif, thus suggesting that ADAMTS2 may also be potentially involved in angiogenesis [123]. The domain and sequence similarities between ADAMTS2 and other anti-angiogenic family members such as ADAMTS1 and ADAMTS8 also suggest a possible role in angiogenesis.

Based on this hypothesis, Dubail *et al*. studied the involvement of ADAMTS2 in angiogenesis (Table 2). Their studies demonstrated that ADAMTS2 inhibited VEGF-stimulated EC proliferation, including both human microvascular endothelial cells (HMVEC) and human umbilical vein endothelial cells (HUVEC). This effect was shown to be specific to ECs as proliferation of either smooth muscle cells or fibroblasts was not affected. Additionally, ADAMTS2 was also shown to induce apoptosis and prevent the formation of capillary tube-like structures in HUVEC. Further, it also prevented the assembly of embryonic stem cells into vascular structures within embryoid bodies upon VEGF stimulation [71].

The authors also demonstrated pronounced morphological changes with disorganized cytoskeleton, numerous blebbing cells and ultimately death mostly by anoikis. An analysis of possible mechanism of anoikis revealed that binding of ADAMTS2 to endothelial cell surface resulted in phosphorylation of Erk1/2. Nucleolin, a nucleolar protein involved in transcriptional control of rRNA genes was identified as the cell surface receptor of ADAMTS2. However, the authors could not demonstrate the functional significance of this receptor due to lack of specific blocking antibodies to the receptor [71].

Using a choroidal neovascularization model, Dubail *et al*. showed that there was a significant increase in new blood vessels in *ADAMTS2* knockout mice compared to wild-type animals (Table 1) [71]. In addition, ADAMTS2-overexpressing HEK293-EBNA cells drastically reduced tumor growth when grafted subcutaneously into nude mice. A dense network of blood vessels was observed in parental tumors while ADAMTS2 overexpressing tumors were whitish with numerous necrotised areas. Similar results were obtained using the catalytically inactive form of protein, although to a lesser extent, suggesting that catalytic activity of the protein is dispensable for its anti-angiogenic function.

In order to delineate the particular domains responsible for this function, authors evaluated two constructs, one lacking the central region including the disintegrin-like domain, the first TSR and the cysteine-rich region and the other lacking the *C*-terminal domains after spacer region, in their ability to modulate *in vivo* tumor growth. Both constructs contained at least one TSR domain suggesting that the ancillary domains, most likely the TSR is responsible for the anti-angiogenic function of the molecule [71]. Further studies are required to precisely identify the regions responsible for mediating this anti-angiogenic role as well as the clinical relevance of this pro-collagen proteinase. In addition, it is necessary to analyze the expression of ADAMTS2 in various human cancers and to analyse whether the vascularization status of these cancers is affected in anyway by this ADAMTS family member. Thus, ADAMTS2 is an example of a family member which suppresses tumor growth through inhibiting angiogenesis.

## 4. ADAMTS4

ADAMTS4 also known as aggrecanase-1 was first purified from IL-1 stimulated bovine nasal cartilage conditioned media and was subsequently cloned as an 837 amino acid protein [32,124]. *ADAMTS4* is located at 1q21-23. The protein is well-characterized for its role in aggrecan cleavage and consequently its involvement in articular cartilage degradation in arthritis [32,33]. However other substrates have been identified in recent years such as versican [23] and brevican [34], thus the protein is now referred to with a more appropriate name of hyalectanase or proteoglycanase.

### 4.1. Structure and Processing

ADAMTS4 like other members is also a secreted protein with a long signal peptide, a prodomain that maintains enzyme latency with a probable cysteine switch at Cys^194^. The catalytic domain has a zinc binding motif similar to that found in the matrix metalloproteinases (MMPs) and ADAMs, which is followed by a disintegrin-like domain. The protein is unique in having a single central TSR motif closely related to ADAMTS1 TSR (Figure 1) [32]. Enzyme latency is removed by furin mediated cleavage of ADAMTS4 within trans-Golgi network by means of multiple consensus sites located between amino acids 208–215, at the end of the prodomain [32,125]. In addition to this, ADAMTS4 undergoes either autocatalytic cleavage or cleavage by glycophosphatidyl inositol-anchored membrane type-4 matrix metalloproteinase at its *C*-terminus. Cleavages between Lys^694^-Phe^695^ and Thr^581^-Phe^582^ resulted in the release of two truncated isoforms of 53 kDa and 40 kDa that lack the *C*-terminal cysteine-rich and spacer domains [126,127]. No cleavage at the *N*-terminal region of the protein has been reported so far. However, preliminary data in our lab has revealed the presence of cleavage events at the *N*-terminal region of the protein, which may result in the release of the *C*-terminal fragments without the catalytic domain [72].

### 4.2. Expression and Regulation

*ADAMTS4* mRNA expression was reported in brain, lung and heart along with very low levels in placenta and skeletal muscle [32]. A recent study using RT-PCR analysis of 15.5–17.5 dpc mouse embryos, new-born and 7-day old animals, showed high expression of *Adamts4* in all stages in the murine kidney, with highest expression in 15.5 dpc embryos [128].

ADAMTS4 enzymatic activity was inhibited by several hydroxamate inhibitors [32]. Additionally, ADAMTS4 was demonstrated by several groups to be inhibited by tissue inhibitors of matrix metalloproteinases (TIMPs), most potent among them being TIMP-3 followed by TIMP-1, TIMP-2 and TIMP-4 respectively [129,130,131].

ADAMTS4 is also regulated by several cytokines and growth factors. Upon stimulation by IL-1, TNF-α or retinoic acid, an increase in aggrecanase activity and mRNA levels, without an increase in protein levels was observed [132,133]. In prostatic stromal cultures, TGF-β treatment caused an increase in *ADAMTS4* expression [42]. Another study also reports the potential involvement of NFATp and Runx2 in regulating ADAMTS4 expression transcriptionally [134]. Besides these, ADAMTS4 is also regulated by the inflammatory cytokine interferon-γ (IFN-γ), in macrophage-rich areas of atherosclerotic plaques [135].

Another level of transcriptional regulation was alternative splicing of *ADAMTS4* observed in human osteoarthritic synovial cocultures, resulting in a protein product with a completely different *C*-terminal spacer domain, which may have relevance in altered substrate specificity [136].

### 4.3. Function

ADAMTS4 has been shown to have diverse functions ranging from ovulation [137], modulation of neural plasticity [138,139] and osteoarthritis [140,141,142]. Studies with *Adamts4*-null mice disclosed that these mice were phenotypically normal and showed no protection against aggrecan degradation in inflammatory or surgically induced arthritis in mice, indicating that *ADAMTS4* is not the main aggrecanase in mouse arthritis (Table 1 and Table 2) [38,44]. Recently many other ECM proteins have been shown to be substrates of ADAMTS4 including matrilin [35], hevin [36] and reelin [37], the latter two being enriched in the brain. This could mean that ADAMTS4 may have other as yet undiscovered functions in development. Indeed, a recent report implicates ADAMTS4 to have redundant and essential roles in perinatal kidney development [128].

### 4.4. Angiogenesis and Cancer

Recent studies suggesting the potential involvement of ADAMTS4 in angiogenesis have emerged (Table 2). Clues pointing to such a hypothesis stemmed from the observation that *ADAMTS4* is closely related phylogenetically to the anti-angiogenic *ADAMTS1*. Peptides from the TSRs of ADAMTS1 and ADAMTS8 have been shown to contain anti-angiogenic properties [12,143]. ADAMTS4 TSR shares strong sequence similarities with ADAMTS1. A novel anti-angiogenic peptide derived from the ADAMTS4 TSR termed adamtsostatin-4 has been identified through a bioinformatics approach. Adamtsostatin-4 inhibited HUVEC proliferation with a biphasic response and also inhibited VEGF-induced HUVEC migration [73].

An early report by Kahn *et al*. indicated an increase in *ADAMTS4* mRNA expression in response to VEGF stimulation and intense mRNA *ADAMTS4* expression in the vascular endothelium [144]. Recently Hsu *et al*. reported that *ADAMTS4* is expressed in HUVEC and human dermal microvascular endothelial cells (HuDMEC). Using a truncated recombinant ADAMTS4 protein that lacks *C*-terminal regions of the protein, the authors demonstrated that ADAMTS4 reduced/inhibited HuDMEC differentiation on Matrigel as well as migration in a scratch wound healing assay without affecting cell proliferation and viability [74]. Like ADAMTS1, ADAMTS4 also seemed to exhibit its anti-angiogenic function via binding and sequestering VEGF and preventing VEGFR2 signalling as observed by the loss of VEGFR2 phosphorylation [79].

Many of ADAMTS4’s substrates are proteoglycans abundantly present in ECM. Hence, it is possible that this multi-domain metalloproteinase may play a role in tumor angiogenesis and tumor progression. Increased expression of *ADAMTS4* was observed in several human cancers such as breast [91], head and neck squamous cell carcinoma [92] and human glioblastoma [93]. In Ewings sarcoma, high expression of ADAMTS4 is a potential tumor marker [94]. Preliminary data in our lab also indicates widespread expression of ADAMTS4 protein in human cancers [72]. Using a syngeneic mouse tumor implant melanoma model, we demonstrated that catalytically-active full-length ADAMTS4 promoted melanoma tumor growth. In contrast, the *C*-terminal ancillary regions and the full-length protein lacking catalytic activity inhibited tumor growth [72]. ADAMTS4’s involvement in tumor angiogenesis is closely linked to its catalytic activity. Thus, ADAMTS4 is similar to ADAMTS1 in having both pro- and anti-tumorigenic activities generated from its various isoforms. Further investigations are required to clarify the contribution of this metalloproteinase in human cancer.

## 5. ADAMTS5

ADAMTS5, also known as ADAMTS11, aggrecanase-2 and *implantin* in mice, is one of the well-studied metalloproteinase in ADAMTS family (Reviewed in [145,146]) due to its role as the major aggrecanase in cartilage degradation in arthritis [38,44]. The human gene is located on chromosome 21q21.3. Small chemical inhibitors of this metalloproteinase have been identified based on the structure of its catalytic domain [147,148,149]. Recent studies have shed light on its role beyond cartilage degradation, revealing its role in development and other pathological conditions including cancer.

### 5.1. Structure and Processing

ADAMTS5 is a 100 kDa secreted matrix metalloproteinase and possesses two TSRs, a central TSR (first TSR) and a *C*-terminal TSR (second TSR) (Figure 1). Like all other ADAMTS members, ADAMTS5 is secreted due to its *N*-terminal signal peptide [39]. The enzyme latency is maintained by its prodomain, which gets cleaved extracellularly. This is different from ADAMTS1 and ADAMTS4 whose processing takes place in trans-golgi network [40,125,150]. However, the processing is also not at the cell surface as in the case of ADAMTS9 [151]. The proADAMTS5 is activated by pro-protein convertases such as furin and PC7 [40].

ADAMTS5 is known to undergo autocatalytic cleavage at the *C*-terminal region resulting in two shorter *N*-terminal fragment isoforms of 45 and 60 kDa [152]. The ancillary domains of this multi-domain proteinase are known to play an important role in localization and substrate specificity [153]. With respect to its aggrecanase activity, it was found that the full-length proteinase has the maximum activity and metalloproteinase domain with just the disintegrin domain loses its catalytic function. Loss of other *C*-terminal ancillary domains reduced the aggrecanase activity considerably [152,153]. Thus the ancillary domains are also referred to as an exosite that helps in substrate binding and catalytic function [146].

### 5.2. Expression and Regulation

Human *ADAMTS5* showed high mRNA expression in brain, intestine, spleen, placenta, uterus, ovary, mammary gland. Fetal tissues showed high expression of human *ADAMTS5* in kidney, spleen and lung. Mild expression was also observed in adult heart, bladder and fetal heart [49]. Detailed expression pattern of *Adamts5* in mouse was studied by McCulloch *et al.* using β-gal staining in *Adamts5* knock-in mice, where a LacZ cassette has been inserted in *Adamts5* locus [154]. *Adamts5* expression is predominantly observed in the neuromuscular structures suggesting its critical role in degrading the proteoglycan—versican which is abundant in such tissues. Early embryonic expression was mainly noted in floor plate, brain and nervous system. In adult tissues, expression was found in various organs such as heart, kidney, lungs *etc*. The expression is mainly restricted to smooth muscles in these organs. In central and peripheral nervous system, expression is in dorsal root ganglia and Schwann cells [154].

ADAMTS5 is regulated at multi levels such as pro-domain cleavage, enzyme glycosylation, mRNA expression, epigenetic modification, *C*-terminal processing, microRNA based inhibition, endogenous inhibitors *etc*. [146,155]. Since this major aggrecanase is implicated in degenerative diseases such as arthritis, its expression in the cartilage and surrounding tissues have been extensively studied. This includes cartilage and synovial tissue. The regulation of this metalloproteinase in presence of inflammatory cytokines such as IL-1α, IL-1β and TNF-α is well studied.

Up-regulation of *ADAMTS5* transcript was observed upon IL-1α, IL-1β and TNF-α stimulation in bovine or human chondrocytes [156,157,158]. However, several other studies have shown that there is no change in the transcript level of *ADAMTS5* upon treatment with IL-1α, IL-1β and TNF-α [159,160,161]. This discrepancy may be due to various reasons such as variation in cell or tissue origin, cell culture conditions, primary or transformed cells used in the experiment *etc*. In addition, fibroblast growth factor 2 (FGF-2) is also known to down-regulate ADAMTS5 [162,163]. Recently, it has been reported that ADAMTS5 undergoes endocytic clearance in normal articular cartilage through low-density lipoprotein receptor-related protein-1 [164]. This is the first receptor reported for ADAMTS5 and is most likely interacts through TSR1/Spacer region of ADAMTS5.

### 5.3. Function

Knockout studies have shown that *ADAMTS5* is the major aggrecanase in osteoarthritis in mice (Table 1) [44,47]. However, not much is known about its physiological role. Recently, more functions are being attributed based on its proteoglycanase function. Its role in degrading proteoglycan versican is being investigated thoroughly due to its complementing expression pattern with versican and its knockout phenotype of syndactyly in mice [45,154]. Didangelos and colleagues have shown the importance of this proteoglycanase in the regulation of proteoglycan turnover and lipoprotein retention in atherosclerosis [162]. They observed a down-regulation of this metalloproteinase in the atherosclerotic aortas of apolipoprotein E null mice (apoE^−/^^−^) and this coincided with the accumulation of biglycan and versican resulting in the retention of low density lipoprotein. ADAMTS5 has been shown to help in dermal wound healing by degrading pericellular aggrecan and activating pro-fibrotic TGF-β1 signaling in fibroblastic cells [46]. Another independent study by Hattori *et al.* also confirmed its role in wound healing which reported the importance of pericellular versican turnover by ADAMTS5 and in regulating fibroblast to myofibroblast transition [165]. These studies clearly indicate that ADAMTS5 is a key player in various physiological and pathological processes involving proteoglycan turnover.

### 5.4. Angiogenesis and Cancer

ADAMTS5 contains two TSRs—central TSR (first TSR) and *C*-terminal (second TSR). Our lab was first to demonstrate that the first but not the second TSR of ADAMTS5 is anti-angiogenic *in vitro* [76]. We showed that recombinant first TSR inhibited *in vitro* EC tube formation, proliferation and induced apoptosis. Recently, we further demonstrated that ADAMTS5 is an endogenous angiogenesis inhibitor both *in vitro* and *in vivo* (Table 2) [75,166]*.* Overexpression of ADAMTS5 in B16 mouse melanoma suppressed primary tumor growth. This tumor growth suppression correlated with decreased tumor vasculature, reduced tumor cell proliferation and enhanced tumor cell apoptosis as shown by immunohistochemistry. Thus, most likely, ADAMTS5 is bringing about tumor growth suppression through inhibiting angiogenesis. This tumor growth mitigation was independent of its proteoglycanase function. Using structural functional domain mapping studies, we showed that TSR1 confers ADAMTS5 with its anti-angiogenic function. Furthermore, overexpression of ADAMTS5 leads to down-regulation of key angiogenic growth factors such as VEGF, placenta growth factor (PlGF), and platelet-derived endothelial growth factor (PD-ECGF) in the tumor milieu.

*ADAMTS5* is reported to be down-regulated in breast cancer by Porter *et al.* (Table 3) [91]. They compared the mRNA level of all the *ADAMTSs* in malignant breast cancer tumors and non-neoplastic mammary tissues and showed that *ADAMTS5* was down-regulated in malignant breast cancer tumors. Another study has shown that *ADAMTS5* transcript was below detectable limit in prostate cancer cell lines PC3, DU145 and LnCaP [42]. It is interesting to note that while there was low expression of *ADAMTS5* in prostate cancer cell lines, the normal prostate stromal cells expressed high levels of *ADAMTS5*. *ADAMTS5* has also been reported to be hypermethylated in colorectal cancer [95]. So, all these studies suggest a possible tumor suppressor function of *ADAMTS5*, which may be a consequence of its ability to inhibit tumor angiogenesis.

However, other reports indicated that *ADAMTS5* is up-regulated in glioblastoma and may help in glial cell invasion [41,93]. The up-regulation of this proteoglycanase in this brain cancer can be attributed to its brevicanase function. Brevican, the brain specific proteoglycan, is abundant in normal adult brain and is thought to be essential for the integrity of brain ECM [167]. In order to invade through the brevican rich ECM, glial cells need to digest this proteoglycan and ADAMTS5 helps in attaining this function. Hence, ADAMTS5 might have different roles in different cancers depending on its substrate availability in different tissues. Nevertheless, its anti-tumorigenic activity is most likely mediated through its anti-angiogenic activity. Further investigations are required to clarify its role in different human cancers.

## 6. ADAMTS8

ADAMTS8, also known as METH-2, is an 890 amino acid multi-domain protein comprising of two TSRs similar to ADAMTS5 [12,168]. Mouse *Adamts8* is located on chromosome 9 and human *ADAMTS8* on chromosome 11q25 [168]. This metalloproteinase can be classified under the sub-group of proteoglycanases [4].

### 6.1. Structure and Processing

ADAMTS8 is secreted to the ECM through its *N*-terminal signal peptide. It also possesses a prodomain that most likely helps it to maintain enzyme latency. There is a furin cleavage site “RTKR” between prodomain and the metalloproteinase domain. The metalloproteinase domain is characteristic with a zinc binding motif “HTLAHELG”. The ancillary domains include a disintegrin-like domain, two TSRs, cysteine rich domain and spacer domain (Figure 1). Even though the mature protein form has a theoretical molecular weight of 75 kDa, 95 kDa size was observed in SDS-PAGE indicating possible glycosylation of this metalloproteinase [49]. Auto-catalytic or other proteinase mediated cleavage was also observed resulting in shorter isoforms of this metalloproteinase.

### 6.2. Expression and Regulation

*ADAMTS8* was found to be expressed mainly in the adult human tissues such as lung, brain, heart and placenta by Northern blot [12]. Comparing to *ADAMTS1*, the expression of *ADAMTS8* was restricted and less abundant. In fetal tissues, *ADAMTS8* was expressed abundantly in lung and moderately in brain and kidney. Expression was absent in many primary cell lines which were of endothelial origin, dermal fibroblasts and vascular smooth muscle cells. Out of several cancer cell lines probed so far, only SW480—a colon carcinoma cell line expressed *ADAMTS8* [12]. A more detailed human *ADAMTS8* transcript expression pattern analysis was performed by Collins-Racie *et al.* using a multiple tissue expression (MTE) array. Highest expression of ADAMTS8 was observed in lung, aorta and fetal heart. Mild expression was seen in appendix and brain. Similar to *ADAMTS5*, *ADAMTS8* also showed narrow expression limited to few tissues. In addition, *ADAMTS8* was expressed in normal and osteoarthritic human cartilage [49].

### 6.3. Function

ADAMTS8 was first discovered together with ADAMTS1 as a novel TSR domain-containing endogenous angiogenesis inhibitor by Iruela-Arispe’s lab [12]. Subsequently, ADAMTS8 was found to cleave aggrecan at Glu^333^-Ala^334^, and hence classified as an aggrecanase (Table 1) [49]. No description on its ability to cleave other substrates in ECM has been reported up to now.

### 6.4. Angiogenesis and Cancer

Like ADAMTS1, ADAMTS8 is also a potent endogenous angiogenesis inhibitor (Table 2). ADAMTS8 inhibits endothelial cell proliferation in a reversible fashion. This inhibition was also specific to cells of endothelial cell origin as fibroblasts and smooth muscle’s proliferation rate was not affected upon ADAMTS8 treatment [12]. Using CAM assay and Matrigel plug assay, Vazquez *et al.* showed that ADAMTS8 suppresses angiogenesis *in vivo.* It is noteworthy that the inhibitory effect of ADAMTS8 was in nanomolar range, more potent than the endogenous angiogenesis inhibitor TSP1 [12]. 

Several reports have emerged about *ADAMTS8* being down-regulated or silenced in various cancers (Table 3). First clue of *ADAMTS8* being a possible tumor suppressor gene surfaced upon gene expression analysis using microarray of primary non-small-cell lung carcinomas (NSCLC) *versus* normal lung tissue [96]. Further studies clearly demonstrated that *ADAMTS8* was epigenetically silenced in primary non-small-cell lung carcinomas (NSCLC) [97]. A dramatic reduction of ADAMTS8 expression in lung cancer was observed in 23 paired normal and lung cancer tissues. Moreover, immunohistochemical studies showed that *ADAMTS8* was indeed down-regulated in NSCLC. *ADAMTS8* expression suppression was a result of *ADAMTS8* promoter methylation and not homozygous deletion or allelic imbalance [97]. Expression of *ADAMTS8* transcript using quantitative RT-PCR in human brain tissues showed that *ADAMTS8* was down-regulated in brain tumors compared to normal brain tissue samples [98]. Using real-time PCR, Porter *et al.* showed that *ADAMTS8* is also down-regulated in breast carcinomas compared to non-neoplastic mammary tissue [91]. In addition, they showed that *ADAMTS8*, along with *ADAMTS15*, are novel predictors of survival in breast cancer patients [169]. These studies suggest that ADAMTS8 is a negative regulator of angiogenesis and cancer. Nevertheless, the possible signaling pathway or receptor through which ADAMTS8 mediates its angiostatic function is unknown and awaits future studies.

## 7. ADAMTS9

ADAMTS9 contains 15 TSRs and a GON-1 domain at the *C*-terminus [50,170]. GON-1 domain shares a high homology with *Caenorhabditis elegans gon-1* gene involved in gonadal development [171]. Thus, *ADAMTS9* is an orthologue of *gon-1* gene of *C. elegans. ADAMTS9* is located on chromosome 3p14.2-14.3 [170]. This metalloproteinase also belongs to the subgroup of proteoglycanases as it has the identical catalytic active site as that of ADAMTS1 and can cleave versican and aggrecan (Table 1) [50].

### 7.1. Structure and Processing

The predicted molecular weight of ADAMTS9 is 216 kDa. ADAMTS9 has a secretory signal peptide and a prodomain comprising of 5 furin cleavage sites [50]. Full-length ADAMTS9 contains 14 *C*-terminal TSRs separated by two linkers and contains the highest number of TSRs in ADAMTS family, similar to ADAMTS20 (Figure 1) [50]. Following the loss of signal peptide and entry into the secretory pathway, ADAMTS9 undergoes prodomain cleavage by pro-protein convertases (e.g., furin). However, unlike ADAMTS4, this prodomain processing occurs outside the cytoplasm—on the cell surface [151]. Cell surface processing of pro-ADAMTS9 is mediated by the chaperone GRP94/gp96 [151]. ADAMTS9 also undergoes post-translational modifications such as glycosylation [50]. Unlike other ADAMTSs such as ADAMTS1, ADAMTS4 or ADAMTS5, prodomain cleavage leads to reduced versicanase activity rather than enhancing the catalytic function [1].

### 7.2. Expression and Regulation

Since Northern blotting could not detect any *ADAMTS9* in fetal and adult human tissues, RT-PCR was employed to study the expression of this proteinase. Highest expression of *ADAMTS9* was observed in human adult tissues such as heart, placenta and skeletal muscle [50]. Low expression was observed in spleen, thymus, prostate and small intestine. *ADAMTS9* was also expressed in testis and ovary, thus revealing its possible role in gonadal development which can be attributed to its GON-1 domain [50]. Detailed spatial and temporal expression pattern analysis of *Adamts9* during embryogenesis of mice was revealed through *in situ* hybridization [172]. At 7.5 dpc, expression of this metalloproteinase is in the decidual tissue and parietal endoderm. At 9.5 dpc, expression of *Adamts9* is observed in mesodermal tissues such as heart. From 11.5 dpc, *Adamts9* transcript is present abundantly in craniofacial region and diaphragm. Post 13.5 dpc, expression is observed in developing organs such as lung, kidney and bone. The expression was also very abundant in vascular endothelium such as capillaries [172]. *Adamts9* knockout mouse is embryonic lethal and die before gastrulation suggesting a quintessential role of this metalloproteinase in embryogenesis (Table 1) [51,52]. Using *Adamts9^+/^^−^* mice which had a LacZ cassette knock-in in one of the *Adamts9* alleles, it was shown that this gene is expressed in the cardiovascular system and its absence resulted in various cardiac and aortic anomalies in a haploinsufficient manner [53].

*ADAMTS9* is transcriptionally regulated in human chondrocytes upon treatment by catabolic agents such as IL-1β and TNF-α. Out of all the *ADAMTS* aggrecanases, *ADAMTS9* showed the highest up-regulation [173]. IL-1β mediated up-regulation of *ADAMTS9* was attributed to the presence of NFAT binding site in the promoter region of *ADAMTS9* gene [174]. TNF-α also up-regulated *ADAMTS9* in a retinal pigment epithelium derived cell line ARPE-19 [175].

### 7.3. Function

Many reports of ADAMTS9 as a proteoglycanase have surfaced in recent years. ADAMTS9’s versicanase activity was shown to be important in cardiac development and allostasis. *Adamts9* haploinsufficiency in mice resulted in a defective myocardium and aortic wall as a result of accumulation of versican [53]. It is also becoming evident that ADAMTS members of the proteoglycanase sub-family act cooperatively and synchronously in maintaining ECM turnover. The role of *Adamts9* in mouse palatogenesis during development was established using double haploinsufficient *Adamts9^+/^^−^* and *Adamts20^+/^^−^* mice [51]. Due to reduced versican degradation, there was impairment in proliferation of palate mesenchyme cells resulting in delayed palate closure. *Adamts9* also regulates interdigital web regression cooperatively with *Adamts5* and *Adamts20* during mouse embryonic development. Inter-digital web regression involves apoptosis and removal of web ECM which is rich in versican. *Adamts9*, together with other versicanases in ADAMTS family such as *Adamts5* and *Adamts20*, cooperatively maintains versican proteolysis during web regression [45]. *Adamts9* and *Adamts20* have also been reported to play a vital role in melanoblast development [176]. These functions of ADAMTS9 are mainly attributed to its versican degradation ability. In addition, ADAMTS9 is also important in degrading the cartilage ECM aggrecan. Up-regulation of ADAMTS9 transcript was observed in immortalized chondrocyte cell line C-28/I2 upon cytokine stimulation involving a mix of IL-1β, TNF-α and interferon-γ. Knockdown of *Adamts9* expression using lentiviral shRNA in 3D chondrocyte culture resulted in an increase in matrix deposition and decrease in aggrecan degradation [177], suggesting an importance of *Adamts9* in cartilage breakdown during pathological conditions such as arthritis. Thus, it is clear that ADAMTS9, in cooperation with other proteoglycanases such as ADAMTS5 or ADAMTS20, plays important roles during embryogenesis, adult physiology and pathology.

### 7.4. Angiogenesis and Cancer

High expression of ADAMTS9 in vascular endothelium suggested its role in angiogenesis [172]. The presence of 15 TSRs also suggested a possible role in inhibiting angiogenesis. Using *Adamts9^+/^^−^* mice, Apte’s group showed that this metalloproteinase is an endogenous angiogenesis inhibitor both in physiological and in pathological setting (Table 2). They observed an increase in corneal neovascularization and tumor vascularization in *Adamts9^+/^^−^* mice compared to wild type mice [52]. Knockdown of *Adamts9* in cultured endothelial cells using siRNA, enhanced capillary network formation and migration, whereas overexpression resulted in suppression of tube formation. This action was not cell line specific as endothelial cells from different sources showing same phenotype suggest a cell-autonomous role for ADAMTS9 in inhibiting angiogenesis [52]. Since ADAMTS9 shares a very high sequence similarity with the anti-angiogenic ADAMTS1, they investigated if the mechanism of ADAMTS9’s angiostatic function is similar to that of ADAMTS1. Surprisingly, even though ADAMTS1 and ADAMTS9 have identical catalytic active site sequence, ADAMTS9 does not cleave TSP1 and TSP2 nor does it bind to and sequester VEGF. This suggests that unique ancillary domains of each member of the ADAMTS family influence its substrate recognition and catalytic activity. It is also quite intriguing to note that the anti-angiogenic function is proteolytic dependent since the catalytically inactive ADAMTS9 loses its anti-angiogenic function [52]. This clearly shows that ADAMTS9 has a proteolytic dependent anti-angiogenic mechanism different from ADAMTS2 and ADAMTS5.

When *ADAMTS9* was mapped to chromosome 3p14.2 it was suspected to be involved in tumor suppression as it shared the same region that was involved in chromosomal translocations in common hereditary renal cell carcinomas [170]. Down-regulation of *ADAMTS9* transcript in malignant breast cancer tissue compared to the non-neoplastic mammary tissue was also observed [91]. The first report of ADAMTS9 being a tumor suppressor was reported in esophageal squamous cell carcinoma (ESCC) using somatic cell hybridization and critical region analysis (Table 3) [99]. *ADAMTS9* was down-regulated in ESCC cell lines and primary tumor tissue samples. Epigenetic silencing through promoter methylation was the primary reason for this down-regulation. Using a similar approach, *ADAMTS9* was also shown to be down-regulated in nasopharyngeal carcinoma and was significantly associated with lymph node metastasis [100]. Using high-resolution melting analysis for high-throughput analysis, promoter methylation of *ADAMTS9* was evaluated in gastric, colorectal and pancreatic cancer. Outcome in all three cancers reconfirmed the tumor suppressor role of *ADAMTS9* [101].

In ESCC and Nasopharyngeal carcinoma (NPC), *ADAMTS9* functions as a tumor suppressor, inhibiting tumor growth through suppressing angiogenesis [77]. Overexpression of ADAMTS9 suppressed both ESCC and NPC tumor growth *in vivo*. Importantly, knockdown of ADAMTS9 in non-tumorigenic HONE1/chromosome 3 microcell hybrid (MCH) cell line MCH8.12, reverts it back to the tumorigenic phenotype. Overexpression of *ADAMTS9* also correlated with the transcriptional down-regulation of pro-angiogenic factors such as matrix metalloproteinase-9 and VEGF. More recently, Du *et al.* showed that ADAMTS9 is also a tumor suppressor in gastric cancer and its anti-tumor function was also a result of suppressing tumor angiogenesis [102]. Mechanistically, ADAMTS9 was shown to inhibit the oncogenic Akt/mTOR/HIF1α signaling pathway that results in reduced proliferation, induction of apoptosis and inhibition of angiogenesis in gastric cancer. This study demonstrates that ADAMTS9 is a tumor suppressor and regulates cancer partly through Akt pathway independent of its role in angiogenesis. Hypermethylation of *ADAMTS9* also correlated with poor survival of gastric cancer patients. Taken together, *ADAMTS9* is a potent tumor suppressor gene that is epigenetically silenced in a wide range of cancers. ADAMTS9 suppresses cancer both directly through inhibition of cancer cell proliferation, induction of cancer cell apoptosis and indirectly by inhibiting angiogenesis.

## 8. ADAMTS12

ADAMTS12 belongs to the small sub-group of mucin-like domain containing ADAMTSs which includes ADAMTS7 and ADAMTS12 [3,4]. This metalloproteinase was first isolated from cDNA library of human fetal lung tissue [178]. Chromosomal mapping of this multi-domain metalloproteinase reveals its localization on 5q35 [178].

### 8.1. Structure and Processing

Full-length ADAMTS12 has a predicted molecular weight of 177.5 kDa. The domain composition of this metalloproteinase is similar to that of other ADAMTSs except for the following: There is 7 *C*-terminal TSRs arranged in two modules of 3 and 4 separated by a mucin domain (Figure 1). At the *C*-terminal end of this metalloproteinase, there lies a protease and lacunin (PLAC) motif. Like all other ADAMTSs, ADAMTS12 enters the secretory pathway and undergoes prodomain cleavage by furin [178]. After being secreted out of the cell, ADAMTS12 remains bound to the cell surface [55]. The mature ADAMTS12 is 175 kDa, possibly due to post-translational modifications. ADAMTS12 also undergoes *C*-terminal processing resulting in a shorter isoform of 83 kDa. However, this shorter form is not due to the auto-catalytic cleavage but might be a result of cleavage by other cellular proteases [178].

### 8.2. Expression and Regulation

No *ADAMTS12* expression was detected in human adult tissues by Northern blot. In fetal tissues, expression was restricted to fetal lung [178]. Out of several catabolic agents and growth factors tested—TGFα, TGF-β, IL-1α, IL-1β, acidic FGF and EGF, only TGF-β was able to up-regulate *ADAMTS12* in human fetal fibroblast cell line KMST [178]. Recently, *ADAMTS12* was found to be preferentially expressed in highly invasive extravillous cytotrophoblasts that plays a key role in epithelial cell invasion. TGF-β was shown to down-regulate *ADAMTS12* in extravillous cytotrophoblasts whereas IL-1β up-regulates this metalloproteinase *in vitro* [179].

### 8.3. Function

ADAMTS12 was first reported to be important in arthritis because of its ability to bind and degrade the cartilage oligomeric matrix protein (COMP). COMP, also known as thrombospondin 5, is a homopentameric extracellular matrix glycoprotein involved in endochordial ossification and stabilization of the ECM in cartilage. It is a potential biomarker to measure cartilage degradation and in arthritis [180]. Functional genetic assay based on yeast two-hybrid system revealed interaction between COMP and ADAMTS12 [54]. The last *C*-terminal module of 4 TSRs serves as a COMP binding domain for ADAMTS12. ADAMTS12 degrades COMP and thus affects the stability of the cartilage ECM. ADAMTS12 was also observed to be elevated in both rheumatoid and osteoarthritis patient samples compared to the normal cartilage [54]. Two endogenous inhibitors, alpha-2-macroglobulin and granulin-epithelin precursor, are known to inhibit ADAMTS12 mediated COMP degradation [181,182].

### 8.4. Angiogenesis and Cancer

ADAMTS12 inhibits the tube-formation by bovine aortic endothelial cells (BAE-1) in a 3D collagen gel suggesting that ADAMTS12 is anti-angiogenic *in vitro* [55]. This study also showed that the anti-angiogenic function is attributed to the TSR modules present in the ADAMTS12, as the conditioned medium overexpressing the truncated ADAMTS12 lacking all the 8 TSRs could not inhibit tubulogenesis. ADAMTS12 was proven to be an anti-angiogenic metalloproteinase beyond doubt using *Adamts12^−/^^−^* knockout mice in *ex vivo* and *in vivo* models of angiogenesis (Table 2) [56]. *Adamts12^−/^^−^* mice had normal gestation and no apparent defects in growth and fertility (Table 1). However, *Adamts12^−/^^−^* mice showed a greater angiogenic response in the bFGF based Matrigel plug assay compared to wild type mice. The aortic explants from knockout mice also showed an increased sprout density suggesting inhibitory action of ADAMTS12 on new blood vessel formation. Furthermore, overexpression of ADAMTS12 in rat aortic ring inhibited the vessel sprout density. In addition, catalytically inactive mutant of ADAMTS12 (H465Q/E466A) did not abrogate the anti-angiogenic function of this metalloproteinase suggesting a metalloproteinase independent action, which most likely is mediated by TSRs [56].

Initial reports of ADAMTS12 being overexpressed in gastrointestinal carcinomas and a few cancer cell lines such as HeLa, A549 lung carcinoma and Burkitt’s lymphoma (Daudi) cells suggested a tumor supportive role for this metalloproteinase [178]. However, subsequent reports by several groups on ADAMTS12 in cancers suggest otherwise [55,56,103]. Overexpression of ADAMTS12 in Madin-Darby canine kidney (MDCK) cell line suppressed the tumorigenic effects of hepatocyte growth factor (HGF). The anti-tumorigenic function was a result of negative regulation of HGF signaling pathway by ADAMTS12. ADAMTS12 blocked the activation of Ras-dependent ERK pathway. Further, this anti-tumor effect was attributed to ADAMTS12s TSR containing ancillary domains as absence of the ancillary domains lost inhibitory function [55]. Overexpression of ADAMTS12 in A549 lung carcinoma also resulted in the tumor growth suppression in nude mice [55]. *ADAMTS12* is also known to be epigenetically silenced in colon and colorectal cancer cell lines due to the promoter hypermethylation (Table 3). However, surprisingly tumor tissues expressed higher levels of ADAMTS12 compared to normal colon tissue. Further investigation revealed that this discrepancy was due to the overexpression of ADAMTS12 in stromal cells that are in vicinity of the tumor cells [103].

In summary, *ADAMTS12* is a tumor suppressor gene, inhibiting tumorigenesis by directly blocking Ras-dependent ERK pathway in cancer cells as well as indirectly by suppressing angiogenesis through its TSR containing ancillary domains.

## 9. ADAMTS13

ADAMTS13, popularly known as von Willebrand factor cleaving protease (vWFCP), is one unique member of the ADAMTS family that do not belong to any other sub-groups. The human ADAMTS13 gene is located on 9q34.2. vWF is a multimeric glycoprotein that mediates the tethering of platelets to vascular subendothelium during vessel wall damage [183]. ADAMTS13 is of immense therapeutic interest because it is the target gene in thrombotic thrombocytopenic purpura (TTP) [184]. TTP is characterized by intravascular destruction of blood cells such as platelets and erythrocytes which might result in major complications such as anemia, renal failure or neurological dysfunction. TTP is caused due to the decreased ADAMTS13 function as a result of mutations in ADAMTS13 gene or autoantibody production against ADAMTS13 [185].

### 9.1. Structure and Processing

ADAMTS13 has two *C*-terminal CUB domains that are unique to this ADAMTS family member. CUB stands for complement C1r/C1s (complement protein), uEGF (sea urchin protein with EGF-like domain), Bmp1 (bone morphogenetic protein 1) and CUB domain was first observed in these proteins. ADAMTS13 possesses one central TSR and seven *C*-terminal TSRs (Figure 1) [186]. *ADAMTS13* is a large gene spanning 29 exons and encodes a 1427 amino acid long protein of predicted molecular mass of 150 kDa [184]. It is known to undergo alternative splicing resulting in various shorter isoforms [186,187]. The *C*-terminal domains are essential for specificity towards the substrate and its cleavage [188,189].

### 9.2. Expression and Regulation

ADAMTS13 was first isolated from human plasma [58,190]. ADAMTS13 was found to be mainly synthesized and expressed in liver. Hepatic stellate cells are thought to be the primary source of plasma ADAMTS13 [191,192]. Other spliced forms were also reported in placenta, brain, prostate and skeletal muscles [186]. There was moderate expression in heart, kidney and testis [193]. Platelets also produce ADAMTS13 that gets localized on its cell surface and is up-regulated upon platelet activation [194]. ADAMTS13 was also shown to be expressed by astrocytes and microglial cells but not in neurons [195]. Upon spinal cord injury, ADAMTS13 expression was elevated suggesting a role in central nervous system injury response/repair. Endothelial cells showed a constitutive expression of *ADAMTS13* [196].

### 9.3. Function

As explained above, ADAMTS13 is well studied for its role in pathophysiological disorder TTP. ADAMTS13 has only one known substrate—vWF. During vascular injury, vWF helps in bringing together platelets and exposed components of the vascular subendothelium. It is important to note that only vWF has the ability to promote platelet adhesion during high shear rates. vWF is a glycoprotein that can vary in size from being a dimer to concatamer whose size may go up to 15,000 kDa. The efficiency of vWF depends on its length. Longer the vWF, the better is its binding ability. ADAMTS13 helps in regulating the length of vWF by cleaving vWF (Reviewed in [183,185,197]). Further, using *Vwf^−/^^−^* and *Adamts13^−/^^−^* mice, protective role of ADAMTS13 against myocardial infarction was reported [198,199].

### 9.4. Angiogenesis and Cancer

Expression of *ADAMTS13* in endothelial cells suggests an important role for this metalloproteinase in angiogenesis (Table 2). ADAMTS13’s substrate vWF is also known to regulate angiogenesis. vWF knockout mice showed increased angiogenesis and vascularization, suggesting an anti-angiogenic role of vWF. Consistently, knockdown of vWF also increased VEGF mediated angiogenesis [200]. Unlike several other ADAMTS members, vWF cleaving proteinase—ADAMTS13 is reported to be pro-angiogenic [10]. Full-length ADAMTS13 was shown to promote HUVEC tube formation, induce cell proliferation and promote cell migration *in vitro*. Truncated ADAMTS13 lacking the *C-*terminal TSRs lost the ability to do so. Paradoxically, full-length ADAMTS13 suppressed VEGF-mediated angiogenesis. In the presence of VEGF, full-length ADAMTS13 suppressed VEGF-mediated HUVEC proliferation, migration and capillary network formation. ADAMTS13s ability to promote angiogenesis independently (in the absence of VEGF) and its inhibitory effect on VEGF-mediated angiogenesis was attributed to its *C*-terminal TSRs. It was shown that a neutralizing antibody against *C*-terminal TSRs number 5-7 or truncated ADAMTS13 lacking *C*-terminal TSRs abolished the pro-angiogenic function of ADAMTS13 and its ability to suppress VEGF-mediated angiogenesis. Similar to ADAMTS1, ADAMTS13 was shown to bind and sequester VEGF [10]. This suggests that ADAMTS13 might have a similar mechanism as that of ADAMTS1 in inhibiting VEGF mediated angiogenesis [79]. However, further studies need to be undertaken to decipher how ADAMTS13 positively regulate angiogenesis in the absence of VEGF.

One study analyzed the multimeric state of vWF and ADAMTS13 metalloproteinase activity on vWF in a wide range of cancers to understand the role of vWF and ADAMTS13 in dissemination and invasiveness of cancer [104]. Comparing metastatic tumors with the primary solid tumor, there was a strong correlation between metastatic tumors and the multimeric uncleaved vWF in blood plasma. More importantly, the multimeric vWF was shown to be a result of deficiency or functional aberration of ADAMTS13 [104]. This suggests that ADAMTS13 might be involved in suppressing tumor cell invasion. Another study assessed the activity of ADAMTS13 in plasmas of patients with malignant tumors of various origins such as colon, blood, breast *etc*. [105]. Lack of ADAMTS13’s catalytic activity in cleaving vWF was associated with progression of various cancers. Plasma samples from patients with advanced stage of malignancy from different cancers showed reduced ADAMTS13 activity (6–30% activity of the normal plasma) [105]. In contrast to the above studies suggesting an inverse correlation between ADAMTS13 and metastasis, Böhm *et al.* reported that there was no correlation between ADAMTS13 activity and tumor malignancy in brain and prostate cancers (Table 3) [106]. Thus, further studies are required to clarify the relationship between catalytic activity of ADAMTS13 and cancer stages. As vWF is an anti-angiogenic glycoprotein, ADAMTS13’s role in regulating angiogenesis through vWF cleavage warrants further investigation.

## 10. ADAMTS15

*ADAMTS15* was identified by cDNA cloning and is located at 11q25, closely linked to another family member *ADAMTS8* [201]. Amino acid sequence alignments revealed 46% identity with ADAMTS1 and ADAMTS5 and 44% with ADAMTS8 placing it within the same proteoglycanases or angio-inhibitors subgroup [201].

### 10.1. Structure and Processing

ADAMTS15 has a predicted molecular weight of 104 kDa and a domain structure that is comprised of a signal peptide, prodomain, a metalloproteinase domain, a disintegrin-like domain; a central TSR domain, a cysteine-rich region and spacer domain followed by and 2 TSRs at the *C*-terminus (Figure 1). The central TSR within this family is generally characterized by the presence of three Trp residues within a conserved sequence context of 20 amino acids. The third Trp in ADAMTS15 however is replaced by a Tyr [201].

ADAMTS15, like other family members is processed by furin at RAKR^212^, releasing prodomain and rendering the protein free from enzyme latency [201]. ADAMTS15 is activated extracellularly and present in the pericellular matrix. Based on sequence analysis, ADAMTS15 contains three potential *N*-linked glycosylation sites [201]. Molokwu *et al.* have recently reported a potential cleavage within the disintegrin domain resulting in the release of a *C*-terminal fragment approximately 53 kDa in size [202].

### 10.2. Expression and Regulation

In mouse embryo, *Adamts15* is expressed in the developing heart at E10.5 and E11.5 dpc, and in musculoskeletal system from E13.5 through E15.5 dpc. *Adamts15* is also highly expressed in several structures within the adult mouse colon. Analysis of *ADAMTS15* expression in human fetal and adult tissues revealed that expression was evident only in fetal liver and kidney, while none of the adult tissues tested showed any presence of *ADAMTS15* transcript [201]. *ADAMTS15* is found to be one of the genes expressed at basal levels in both prostate cancer cells and the associated stromal cells [42].

*ADAMTS15* was found to be one of the candidate tumor suppressor genes mutated in a small set of 11 colorectal cancer samples including both cell lines and human tumor xenografts (Table 3) [203]. Viloria *et al.* extended this study with a larger panel of 50 colorectal cancer samples and 6 colon cancer cell lines and discovered four new mutations, three in cancer tissues and one in cancer cell lines all of which could affect normal functioning of this protein [107]. Another report also indicates the presence of a range of *ADAMTS15* somatic mutations in pancreatic cancers from non-silent point mutation to deletions, duplications and substitutions [108].

Unlike other members of the same family such as *ADAMTS1*, *ADAMTS9* and *ADAMTS18*, there was no evidence of epigenetic silencing in tumors in case of *ADAMTS15* [107]. However, it is noteworthy that *ADAMTS15* is located at 11q24.3 in a region of frequent loss of heterozygosity in different tumors especially colorectal cancers [109].

*ADAMTS15* expression has also been shown to be responsive to androgen regulation. *ADAMTS15* was found to contain one androgen response element (ARE) in its promoter and 12 ARE’s within the gene itself using NUBIscan online nuclear receptor binding site search tool. Stimulation of an androgen responsive cell line LnCAP with dihydrotestosterone resulted in marked reduction in *ADAMTS15* expression levels [202].

### 10.3. Function

Similar to other members of proteoglycanase subgroup within the ADAMTS family, ADAMTS15 is able to cleave aggrecan at multiple sites (Table 1) [61]. It also cleaves V1 versican at position E^441^-A^442^ [62].

### 10.4. Angiogenesis and Cancer

ADAMTS15, as mentioned earlier, was recently shown to potentially undergo cleavage within its disintegrin domain to release a *C*-terminal fragment. This fragment closely resembles the anti-angiogenic fragment of ADAMTS1 suggesting a potential role for ADAMTS15 in angiogenesis, although no studies have linked it to angiogenesis thus far (Table 2) [202].

Porter *et al.* have described dysregulated expression of many *ADAMTS* genes in breast cancer. Among which, *ADAMTS15* alone, emerged as a predictor of prolonged event-free survival and was shown to be significantly down-regulated in grade 3 breast carcinomas compared to grades 1 and 2 breast carcinomas [91]. Using a PyMT model, a robust transgenic model of highly metastatic mammary carcinoma that closely resembles human breast cancer of high grade and aggressive disease outcomes, the same group showed that low expression levels of *ADAMTS15* together with high expression of *ADAMTS8* were associated with poor clinical outcome [169]. Overexpression of either *ADAMTS15* or its catalytic active site mutant did not impact cell proliferation or adhesion in two types of breast cancer cells. However, a marked reduction in cell migration was observed in both cell lines [204]. This data suggests that in breast cancer, *ADAMTS15* might be exerting its tumor suppressive function via controlling the interaction of cells with the environment in an enzyme-independent manner [204].

*ADAMTS15* expression in prostatic cancer and associated stromal cells has been previously reported. Cross *et al.* show that, in response to TGF-β stimulation, basal *ADAMTS15* levels dropped in prostatic stromal cells, with a concomitant increase in versican, which is an indicator of poor prognosis in prostate cancer [42]. *ADAMTS15* was frequently mutated in colorectal cancers [107,203]. One of the mutations that caused a deletion of last two TSRs (G849fs mutation) was found to affect the localization of protein, abolishing its ability to be associated with the ECM, which could in turn lead to either loss of normal function or result in the gain of abnormal functions [107]. The authors also demonstrated using two different colon cancer cell lines (HCT-116 and SW-480), that overexpression of ADAMTS15 resulted in reduced colony forming ability as well as a reduced ability to invade. Conversely, siRNA mediated knockdown of the gene caused an increase in clonogenicity and invasive abilities of these cells [107]. Notably, G849fs mutant cells exhibited a reduced effect on either colony formation or tumor cell invasion. However, no effect on motility of either the wild-type or the G849fs mutant overexpressing cells was observed in wound healing assays. Since similar inhibitory effects were also exhibited by a catalytically inactive form of ADAMTS15, the authors conclude that terminal TSRs are responsible for the anti-tumor function of ADAMTS15. Furthermore, overexpression of the G849fs mutant form or knockdown of ADAMTS15 in HCT-116 cells both caused drastic morphological changes in these cells. Cells were larger, flatter and spindle shaped compared to the more round, smaller cells of control or ADAMTS15-overexpressing cells, suggesting the acquirement of invasive capacities [107].

The same authors further went on to show that ADAMTS15 depleted cells showed enhanced *in vivo* tumor growth. Additionally, an immunohistochemical analysis of normal versus tumor colorectal samples showed that ADAMTS15 expression was preferentially located to normal tissues. Moreover, expression of ADAMTS15 inversely correlated with the histopathological grade of colorectal tumors, signifying its utility as an effective differentiation marker [107]. The anti-tumor function of ADAMTS15 correlated with the ability of this protein to modulate Ras-dependent ERK pathway. Tumor cells expressing wild-type ADAMTS15 showed very low level of phospho-ERK. In comparison, cells expressing the G849fs mutant form of *ADAMTS15* retained its ability to phosphorylate ERK in comparison [107]. Thus, the *C*-terminal TSR domains are required for effective inhibition of ERK activity by ADAMTS15. Hence, ADAMTS15 functions as a tumor suppressor in colorectal cancer independent of its metalloproteinase activity.

Thus, expression of ADAMTS15 may protect the organism from cancer development. Whether ADAMTS15 also influences angiogenesis has not been reported. More studies are required to fully understand the importance of this tumor suppressor gene in human cancer and to develop anticancer therapeutics from this protein.

## 11. ADAMTS18

*ADAMTS18* was identified as a novel tumor suppressor gene located at 16q23.1. epigenetically silenced in multiple carcinomas (Table 3) [205]. It is most closely related to *ADAMTS16* with 57% overall identity and 85% identity in their catalytic domains [201].

### 11.1. Structure and Processing

ADAMTS18 is a 135 kDa protein with a domain structure comprising a long signal peptide, prodomain, a metalloproteinase domain, a disintegrin-like domain, a central TSR, a cysteine-rich region, spacer domain, followed by 5 TSR repeats and a protease and lacunin motif (PLAC domain) in the *C*-terminus (Figure 1) [201].

Like all ADAMTSs, ADAMTS18 also possesses a furin cleavage site that releases the mature protein via cleavage of prodomain [201]. In addition, ADAMTS18 is cleaved between Arg775 and Ser776 in the spacer domain by thrombin to release a *C*-terminal 45 kDa platelet active fragment comprising of terminal five TSR domains and PLAC domain [206]. A consensus sequence (NVT) for *N*-glycosylation is found in its catalytic domain [201].

### 11.2. Expression and Regulation

*ADAMTS18* mRNA was found to be widely expressed in all normal human tissues in high amounts [205]. In human fetal tissues, *ADAMTS18* mRNA expression was found in lung, liver and kidney [201]. It has been shown that ECs constitutively express *ADAMTS18* [201,207]. TNF-α induced secretion of *ADAMTS18* in cultured ECs [207].

### 11.3. Function

*ADAMTS18* gene has been recently discovered to be mutated and responsible for Knobloch syndrome, a developmental disorder characterised by occipital skull defect, high myopia, and vitreo-retinal degeneration using a combined exome and autozygome analysis approach [208]. The authors show a strong expression of *Adamts18* mRNA in the lens and retina during mouse embryonic development suggesting a role for ADAMTS18 in eye development [208]. Reports of ADAMTS18 causing oxidative platelet fragmentation and platelet-thrombus degradation, upon thrombin cleavage, suggest that it could have a physiological role in hemostasis [207].

### 11.4. Angiogenesis and Cancer

There has been no study linking ADAMTS18 to angiogenesis (Table 2). Expression analysis of ADAMTS18 revealed that its mRNA expression was dramatically reduced or completely silenced in multiple carcinoma cell line including oesophagus, nasopharynx, stomach, colon, breast, lung and cervix [205]. Another report also identified *ADAMTS18* as a highly down-regulated gene in breast carcinoma as compared to normal tissue irrespective of tumor heterogeneity, type or grade [91]. In comparison, high *ADAMTS18* expression was retained in non-tumor cell lines and in immortalized but non-transformed epithelial cell lines, indicating that this gene is likely to be specifically down-regulated in carcinoma [205]. *ADAMTS18* promoter methylation is also prevalent in a large number of tumor samples of gastric, colorectal and pancreatic origins [110].

Down-regulation of *ADAMTS18* expression was expected to be due to genetic deletion as it is located in chromosome 16q23.1, a region known to be frequently deleted in multiple cancers [209,210,211,212]. Surprisingly, no obvious deletion was detected in cancers with reduced ADAMTS18 expression. Analysis of epigenetic mechanisms of transcriptional control then showed that *ADAMTS18* is regulated via methylation of CpG islands in its promoter and that it is a tumor-specific methylation (Table 3) [205]. However, genetic mutations may also inactivate/activate *ADAMTS18*. Sjobolm *et al*. have reported two missense mutations (R382K and K455T, both within the metalloproteinase catalytic domain) of *ADAMTS18* in 2/11 colon tumors [203]. Another report also identifies *ADAMTS18* mutations in kidney and colorectal cancers [111].

A large fraction of melanomas are known to harbor somatic mutations. The most highly mutated *ADAMTS* gene in melanoma was identified to be *ADAMTS18* with 14 somatic mutations [112]. In the study done by Wei *et al*, effect of *ADAMTS18* mutations on tumorigenic phenotypes was analyzed using six tumor-derived ADAMTS18 mutants (G312E, P452S, C638S, Q904X, Q1002X, and P1035S) and compared with wild-type ADAMTS18. Overexpression of the mutated ADAMTS18 protein in either A375 or Mel-STR melanoma cells (both harbor wild-type ADAMTS18 gene) resulted in higher cell transformation ability while cells overexpressing the wild-type protein behaved similarly with vector-modified cells. In addition, mutant ADAMTS18 overexpression confers a higher proliferation rate to tumor cells *in vitro* under low serum conditions although no growth advantages were observed under normal serum conditions [112]. Mutated *ADAMTS18* also caused reduced adhesion of melanoma cells on laminin-1 and a corresponding increase in cell migration. The authors also show that mutations in *ADAMTS18* affected its cellular localization, causing more of the secreted protein to be retained on the cell surface rather than to be released into the surrounding medium [112]. Using shRNA based knockdown, ADAMTS18 was shown to be essential for melanoma cells to migrate [112]. Xenograft studies in SCID mice revealed that overexpression of mutated ADAMTS18 in Mel-STR cells resulted in increased metastases *in vivo* while overexpression of wild-type protein suppressed metastasis. These results demonstrated an oncogenic role of ADAMTS18 mutants in human melanoma and the *ADAMTS18* mutations are driver mutations. In the same study, wild-type ADAMTS18 showed an assay-specific tumor suppressive effect, suppressing melanoma metastasis *in vivo* but not melanoma cell transformation ability *in vitro* [112]. On the other hand, another study by Jin *et al.* showed that *ADAMTS18* is frequently hypermethylated in tumors and overexpression of wild-type ADAMTS18 inhibits both anchorage-independent and -dependent nasopharyngeal and esophageal carcinoma cell growth using monolayer colony formation and soft agar assays, indicating a tumor suppressor role of this gene [205].

Although ADAMTS18 is a potential tumor suppressor, paradoxically, its mutations seem to have acquired an oncogenic potential in human melanoma. In addition, no function in angiogenesis has been reported. Thus, the roles of ADAMTS18 in different human cancers require further clarification.

## 12. Conclusions

**Figure 2:**
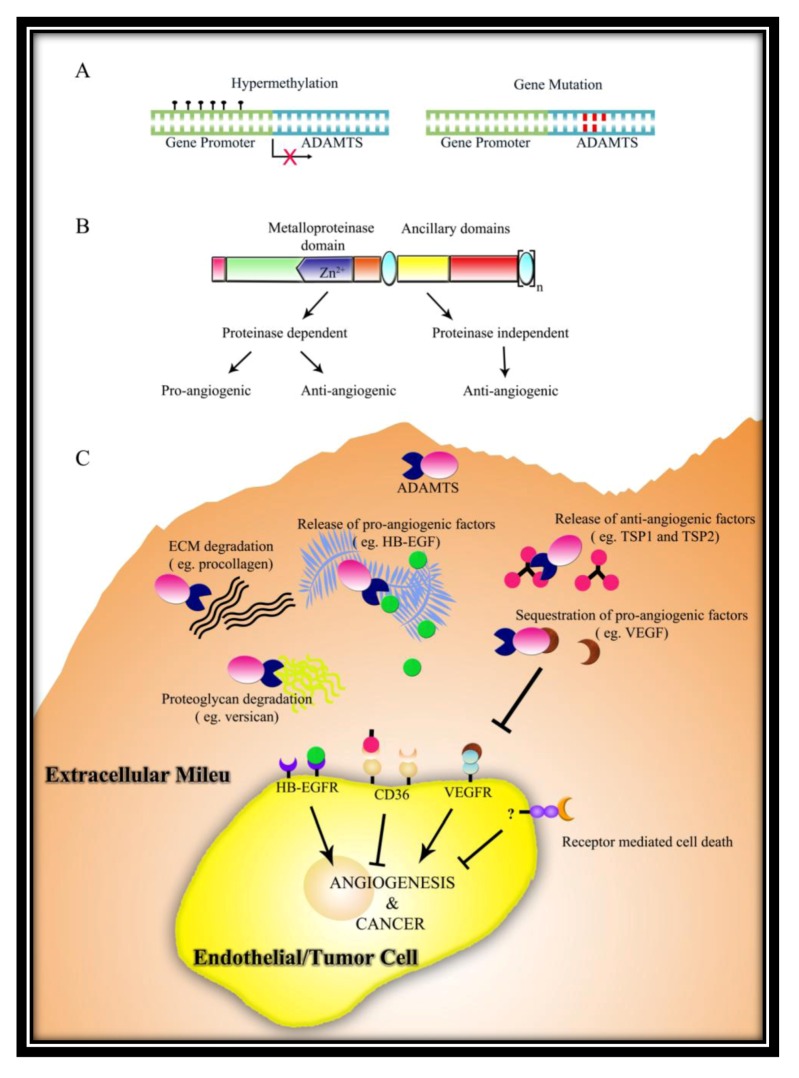
Role of ADAMTSs in angiogenesis and cancer. (**a**) Many ADAMTS members have been shown to be down-regulated in cancer. This down-regulation is mostly through epigenetic silencing (*ADAMTS1*, *ADAMTS5*, *ADAMTS8*, *ADAMTS9*, *ADAMTS12* and *ADAMTS18*). Somatic mutations in ADAMTS gene have also been reported (ADAMTS13, ADAMTS15 and ADAMTS18). (**b**) ADAMTS members regulate angiogenesis and cancer in a complex manner that involves multiple mechanisms. Both proteinase-dependent and -independent anti-angiogenic/anti-tumorigenic mechanisms have been revealed. On the other hand, proteinase-dependent pro-angiogenic/pro-tumorigenic functions have also been reported. (**c**) Illustrations of molecular mechanisms by various ADAMTS members in angiogenesis and cancer. Examples include pro-angiogenic/pro-tumorigenic function through cleaving extracellular matrix substrates such as versican or brevican (e.g., ADAMTS1); help in releasing pro-angiogenic growth factors such as HB-EGF; proteinase-dependent release of anti-angiogenic fragments from ECM (ADAMTS1 releasing TSP1 and TSP2); proteinase-independent sequestration of pro-angiogenic factors (ADAMTS1); TSR-mediated (ADAMTS5) anti-angiogenesis and receptor mediated cell death (ADAMTS2).

For the past few years, a large number of reports have emerged regarding ADAMTS in angiogenesis and cancer (summarized in Figure 2).

Based on the current review, it is clear that most of ADAMTSs are anti-angiogenic or tumor suppressors with a couple of exceptions. However, it is important to note that the anti-angiogenic function of different ADAMTS members differs in their mechanisms of action (Figure 2). The angiostatic function might be broadly classified as proteolytic-dependent or -independent. Proteolytic-independent function of ADAMTS can be a result of its anti-angiogenic TSRs or TSRs together with the ancillary domains involved. Most ADAMTSs discussed in this review are down-regulated in cancers due to promoter hypermethylation. Other ADAMTSs are also reported to be pro-angiogenic and pro-tumorigenic (ADAMTS1, ADAMTS4). A paradox is ADAMTS13 which was shown to promote angiogenesis on its own but inhibit VEGF-mediated angiogenesis. In addition, not all ADAMTS family members regulate cancer through influencing angiogenesis. Since ADAMTSs are ECM metalloproteinases, cleavages of their substrates also might promote cancer cell invasion and metastasis as in the case of ADAMTS1 [84,85]. In addition, ADAMTS9 acts as a tumor suppressor by directly inhibiting tumor cell proliferation and inducing tumor cell apoptosis through inhibition of Akt signaling pathway [102]. Thus, direct impact on tumor cells without the involvement of angiogenesis is also an important mechanism for ADAMTS members to regulate cancer.

Several ADAMTS members share similar physiological functions and act through a cooperative manner in certain physiological processes such as morphogenesis during embryonic development. Similar functional studies using double or triple knockout mice in cancer and angiogenesis may shed light into any cooperative role of ADAMTS members in this area. More mechanistic studies are also needed to fully understand how each member functions in human cancer. It is clear that members of this family are important players in angiogenesis and cancer. ADAMTS proteins or their peptide derivatives can serve as prototypes or targets towards the development of clinically relevant cancer therapeutics.
